# Mesenchymal stem cells enhance the oncolytic effect of Newcastle disease virus in glioma cells and glioma stem cells via the secretion of TRAIL

**DOI:** 10.1186/s13287-016-0414-0

**Published:** 2016-10-10

**Authors:** Gila Kazimirsky, Wei Jiang, Shimon Slavin, Amotz Ziv-Av, Chaya Brodie

**Affiliations:** 1 Mina & Everard Goodman Faculty of Life-Sciences, Bar-Ilan University, Ramat-Gan, Israel; 2 Hermelin Brain Tumor Center, Department of Neurosurgery, Henry Ford Hospital, 2799 W Grand Blvd, Detroit, MI 48202 USA; 3 Hadassah Medical Center, Hebrew University, Jerusalem, Israel

**Keywords:** Newcastle disease virus (NDV), Glioblastoma (GBM), Glioma stem cells (GSCs), Mesenchymal stem cells (MSCs), TRAIL, γ-radiation, Apoptosis, Self-renewal

## Abstract

**Background:**

Newcastle disease virus (NDV) is an avian paramyxovirus, which selectively exerts oncolytic effects in cancer cells. Mesenchymal stem cells (MSCs) have been reported to affect tumor growth and deliver anti-tumor agents to experimental glioblastoma (GBM). Here, we explored the effects of NDV-infected MSCs derived from different sources, on glioma cells and glioma stem cells (GSCs) and the mechanisms involved in their effects.

**Methods:**

The glioma cell lines (A172 and U87) and primary GSCs that were generated from GBM tumors were used in this study. MSCs derived from bone marrow, adipose tissue or umbilical cord were infected with NDV (MTH-68/H). The ability of these cells to deliver the virus to glioma cell lines and GSCs and the effects of NDV-infected MSCs on cell death and on the stemness and self-renewal of GSCs were examined. The mechanisms involved in the cytotoxic effects of the NDV-infected MSCs and their influence on the radiation sensitivity of GSCs were examined as well.

**Results:**

NDV induced a dose-dependent cell death in glioma cells and a low level of apoptosis and inhibition of self-renewal in GSCs. MSCs derived from bone marrow, adipose and umbilical cord that were infected with NDV delivered the virus to co-cultured glioma cells and GSCs. Conditioned medium of NDV-infected MSCs induced higher level of apoptosis in the tumor cells compared with the apoptosis induced by their direct infection with similar virus titers. These results suggest that factor(s) secreted by the infected MSCs sensitized the glioma cells to the cytotoxic effects of NDV. We identified TRAIL as a mediator of the cytotoxic effects of the infected MSCs and demonstrated that TRAIL synergized with NDV in the induction of cell death in glioma cells and GSCs. Moreover, conditioned medium of infected MSCs enhanced the sensitivity of GSCs to γ-radiation.

**Conclusions:**

NDV-infected umbilical cord-derived MSCs may provide a novel effective therapeutic approach for targeting GSCs and GBM and for sensitizing these tumors to γ-radiation.

## Background

Glioblastoma (GBM) remains one of the most malignant disorders in man with an average survival of 14 months despite optimal surgery, radiation therapy and temozolomide, which are considered the standard treatment of choice [1, 2]. Due to the infiltrative/invasive nature of GBM and the complexity of the brain anatomy tumors cannot be completely removed in the large majority of cases [2]. GBMs contain a small population of glioma stem cells (GSCs) that exhibit treatment resistance which prevents a complete eradication of tumors cells and is associated with tumor recurrence [3, 4]. Therefore, new approaches for targeting resistant glioma cells and GSCs are urgently indicated to improve the prognosis of patients with GBM.

One of the novel approaches for the selective targeting of tumor cells is based on the use of oncolytic viruses [5, 6]. These treatments combined specific tumor cell lysis by the viruses together with acting as in situ tumor vaccine [6]. Indeed, the potential use of oncolytic viruses for the treatment of cancer has been reported by several investigators with documentation of long-term survival of patients considered fully resistant to other available anti-cancer modalities [7]. Oncolytic viruses such as herpes simplex virus [8], vaccinia virus [9] and polio virus [10], have been reported as effective and selective therapies in GBM. In addition, there have been a number of reports indicating that Newcastle disease virus (NDV) also acts as an oncolytic virus in a number of tumors including GBM [11–16]. NDV is a well-known poultry virus with anti-neoplastic properties [17]. The preferential oncolytic activity of NDV toward malignant compared to normal cells is not fully understood, but has been attributed in part to reduced interferon secretion by malignant cells in contrast to normal cells [18]. Other mechanisms associated with the anti-tumor activity of NDV were also reported such as activation of the intrinsic death pathway, activation of the endoplasmic eIF2a kinase PERK and caspase 12, and the secretion of tumor necrosis factor alpha (TNF-α) or TNF-related apoptosis-inducing ligand (TRAIL) from the infected tumor cells [7, 19–22]. Indeed, TRAIL has been considered as a promising anti-tumor agent with a strong clinical therapeutic potential [23, 24] and various studies demonstrated selective apoptotic effects of TRAIL on tumor cells including glioma cells and GSCs [25–29].

Administration of oncolytic viruses in clinical trials for GBM involves intratumoral or intravenous injections, which are associated with inefficient virus delivery [30]. One of the alternative approaches that have been explored for efficient virus delivery is the use of stem cells, such as mesenchymal stem cells (MSCs), as delivery vehicles. MSCs exhibit homing abilities to sites of injury, inflammation and tumors [31–34]. Specifically, MSCs have been shown to migrate to sites of experimental GBMs and to deliver cytotoxic compounds that exert anti-tumor effects [31, 32]. MSCs can be obtained from autologous bone marrow (BM) and adipose (AD) tissues [35, 36] or from allogeneic placenta and umbilical cord, which then can be used as “off-the-shelf” cells [37, 38]. These cells can be easily expanded in vitro and used safely for various therapeutic indications [39, 40]. Recent studies suggest that despite sharing similar cell surface markers, MSCs that are derived from different sources exhibit differences in their transcriptome, cytokine profile and biological effects [35]. Therefore, MSCs from various sources can have different therapeutic impacts in specific clinical indications.

In view of the broad and selective anti-cancer properties of NDV [41, 42] and the fact that MSCs migrate actively to tumor sites and cells including cancer stem cells, we investigated the potential therapeutic effect of NDV-infected MSCs on glioma cells and GSCs.

## Methods

### GSC cultures

All human materials were used in accordance with the policies of the institutional review board at Henry Ford Hospital, Detroit, MI, USA. The generation of the GSCs and their characterization were recently described [43–45]. Briefly, GBM specimens were dissociated in 0.05 % Trypsin/EDTA for 4 h at room temperature followed by mechanical dissociation. Cells were maintained in neurosphere medium supplemented with 20 ng/ml epidermal growth factor (EGF) and 20 ng/ml basic fibroblast growth factor (FGF-beta) and were examined for the expression of the stemness markers, CD44, Bmi-1, CD133, Musashi-1, Sox2 and nestin and self-renewal. All the GSCs employed in this study were examined for tumorigenic potential in nude mice or rats as recently reported [43–45].

### Mesenchymal stem cell cultures

Bone marrow (BM)-derived MSCs, adipose tissue (AD)-derived MSCs, and umbilical cord (UC) tissue-derived MSCs were obtained from ScienCell Research Laboratories (Carlsbad, CA, USA) and were characterized and maintained as previously described [46]. The cells expressed CD73, CD90 and CD105 and were negative for CD14, CD34, CD80 and CD45. The different cell types were also examined for their ability to differentiate to osteoblasts, chondrocytes and adipocytes. The purity of all the MSC preparations was over 95 %.

### Co-culture experiments

For the co-culture experiments, MSCs and glioma cells or GSCs were plated in transwell plates with a 0.4-μm filter. In some experiments, conditioned medium of infected MSCs was isolated and administered directly to the GSCs or glioma cells.

### NDV infection

Oncolytic NDV (MTH-68) prepared at the Beit Dagan Institute, Israel was used in all experiments. Cells were infected with different titers of NDV for 2 h, after which the cells were washed three times and incubated with fresh medium.

### Real-time PCR analysis

Total RNA was isolated from cultured cells using QIAzol reagent (Qiagen, Valencia, CA, USA) according to the manufacturer’s protocol. A total of 0.5 μg of RNA was employed to synthesize cDNA by Thermoscript (Invitrogen, Carlsbad, CA, USA) with oligodT primers. Primers, 25 μL of 2× SYBR Green Master Mix (Invitrogen), and 30–100 ng cDNA samples were resuspended in a total volume of 50 μL PCR amplification solution. Reactions were run on an ABI Prism 7000 Sequence Detection System (Applied Biosystems, Foster City, CA, USA). Cycle threshold (Ct) values were obtained from the ABI 7000 software. The following primers were used: NDV: 5’-TCACAGACTCAACTCTTGGG-3’ and 5’-CAGTATGAGGTGTCAAGTTCTTC-3’ as reported [47]. S12 expression was determined for each RNA sample as a control.

### Self-renewal assay

The formation of secondary neurospheres by GSCs was measured in cells that were plated in 24-well plates at a density of 10 cells/well through limiting dilution. The number of neurospheres/well was determined 2 weeks later for ten different wells. Spheres that contained more than 20 cells were scored as described [43–45].

### Cell death assays

Three methods were employed to analyze cell death: (1) measurements of lactate dehydrogenase (LDH) levels in culture supernatants, (2) caspase3/7 activity, and (3) expression of total and cleaved PARP by Western blot analysis. Caspase-3/7 activity in the GSCs was measured using a caspase-3/7 assay kit (Promega, Madison, WI, USA) according to the manufacturer’s instructions.

### Western blot analysis

Western blot analysis was performed as described. Equal loading was verified using an anti-β-actin or tubulin antibodies as described [45, 46].

### TRAIL secretion and neutralization

The concentrations of TRAIL ligand in cell supernatants were measured using a commercial ELISA kit from Diaclone Research (Besancon, France), according to the manufacturer’s instructions. A TRAIL neutralizing antibody (Abcam, Cambridge, MA, USA; 5 μg/ml)), was added in some of the experiments 24 h prior to the addition of the conditioned medium. A corresponding isotype-matched antibody was used as a control.

### Statistical analysis

The results are presented as the mean values ± standard error of the mean (SE). Data were analyzed using analysis of variance or a Student's *t* test with correction for data sets with unequal variances.

## Results

### NDV exerts selective oncolytic effects on glioma cells and GSCs

**Fig. 1:**
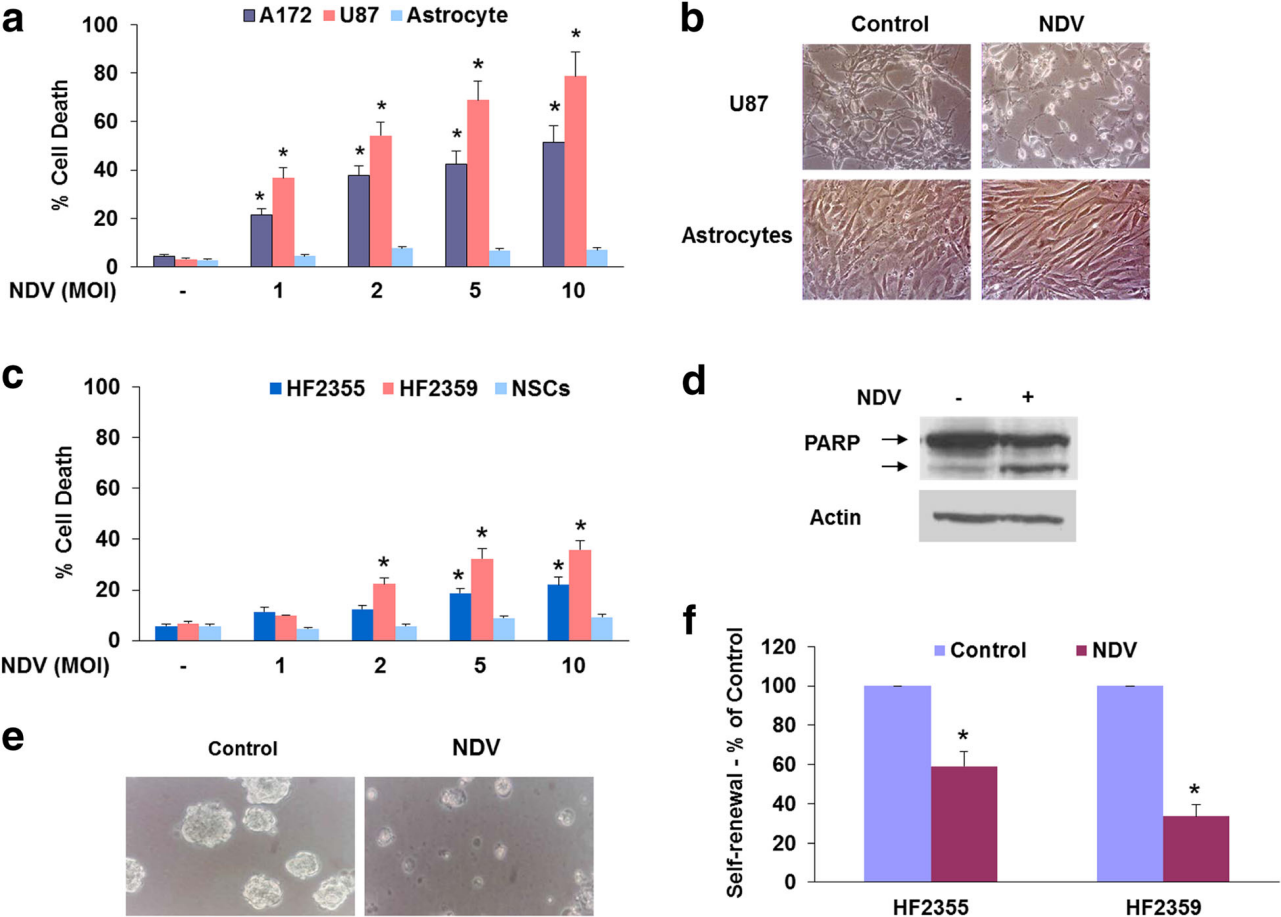
NDV induces a selective cell death in glioma cells and glioma stem cells. The glioma cell lines, U87 and A172 or human astrocytes were infected with different titers of NDV and cell death was determined using LDH release into the culture supernatants after 48 h (**a**). The morphology of U87 cells and human astrocytes was analyzed following NDV infection (2 MOI) using phase contrast microscopy (**b**). Cell death was also analyzed in two GSC cultures and human NSCs using LDH assay (**c**) and in the HF2355 cells using Western blot analysis of cleaved PARP expression (**d**). Infection with NDV induced disaggregation of the GSC spheroids (**e**). The self-renewal of the infected GSCs was determined after 14 days of infection (1 MOI) (**f**). The results are presented as means ± SE and represent three different experiments (**a**, **c**). ^*^ *p* < 0.001 (control vs. infected cells). One representative of three similar experiments is presented (**b**, **d**-**f**). *MOI* multiplicity of infection, *NDV* Newcastle disease virus, *NSC* neural stem cell

We first examined the oncolytic effects of NDV on glioma cell lines and GSCs. Cells were infected with increasing titers of NDV and cell death was examined after 24 and 48 h. As presented in Fig. 1a, NDV induced cell death in both U87 and A172 glioma cell lines already in 1 multiplicity of infection (MOI) and plateau levels were obtained at 5 MOI for both cell lines. In contrast, infection of human astrocytes with 10 MOI of NDV induced only a small degree of cell death (Fig. 1a). Morphological analysis of the infected cells demonstrated similar results - increased cell death in the infected U87 cells with no differences in the cell morphology of human astrocytes (Fig. 1a).

Although NDV has been reported to exert potent oncolytic effects on cancer cells, its effects on cancer stem cells or GSCs has not been described. We therefore examined the oncolytic effect of NDV on GSCs obtained from fresh glioma specimens that were previously described and reported by us [43, 44, 46, 48]. In these studies, we employed the two GSCs HF2355 and HF2359 and examined the effects of NDV infection on the self-renewal and cell death of these cells. We found that NDV induced cytotoxic effects on both GSCs albeit to a different degree (Fig. 1c) as determined by LDH assay and by PARP cleavage for the HF2359 cells (Fig. 1d). For both GSCs, NDV exerted a lower cytotoxic effect compared to the glioma cell lines. Similar results were obtained for an additional two GSCs (data not shown). In contrast, no significant cytotoxic effect was observed in human neural stem cells (NSCs) even at 10 MOI and after 72 h (Fig. 1c).

The cytotoxic effect of NDV was also observed on the stemness characteristics of the GSCs including smaller neurosphere size (Fig. 1e) and inhibition of self-renewal of these cells (Fig. 1f). Using secondary neurosphere formation assay, we found that after 10 days NDV at MOI of 1 significantly decreased the neurosphere size (Fig. 1e) and the self-renewal of the GSCs (Fig. 1f).

### Conditioned medium of NDV-infected MSCs enhances the virus cytotoxic effect

**Fig. 2:**
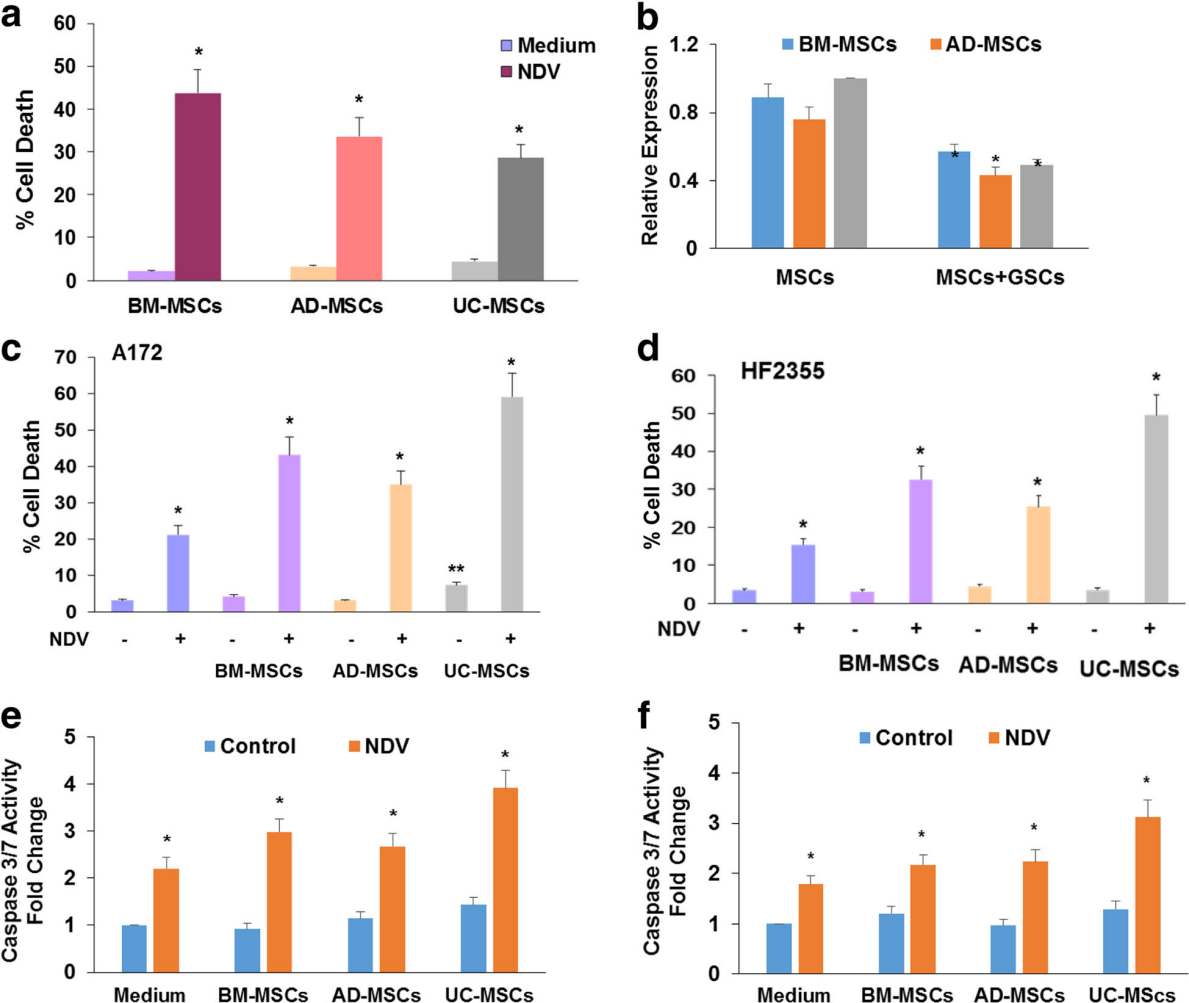
Conditioned medium of NDV-infected MSCs exerts potent cytotoxic effects on glioma cells and GSCs. MSCs derived from BM, AD or UC tissue were infected with NDV (5 MOI) and cell death was determined after 3 days using LDH assay (**a**). MSCs were infected with NDV (2 MOI), washed three times and co-cultured with A172 cells in transwell plates with 0.4 μm for 48 h. The A172 cells were washed three times and the presence of NDV in the cells was determined using RT-PCR (**b**). The A172 cells (**c**, **e**) or HF2355 GSCs (**d**, **f**) were either infected with 2 MOI NDV or incubated with medium conditioned from control or NDV-infected MSCs for 2 days. Cell death was determined using LDH assay (**c**, **d**) or caspase 3/7 activity assay (**e**, **f**) after 24 h. The results are presented as means ± SE and represent three different experiments. ^*^ *p* < 0.001 (control vs. infected cells). *AD* adipose tissue, *BM* bone marrow, *GSC* glioma stem cell, *MSC* mesenchymal stromal cells cell, *NDV* Newcastle disease virus, *NSC* neural stem cell, *UC* umbilical cord

MSCs have been reported to deliver oncolytic viruses to various tumors including glioma [16]. To examine the ability of MSCs to deliver NDV to glioma cells we first analyzed the infection of the different MSCs by NDV. For these experiments, we employed MSCs derived from BM, AD and umbilical cord (UC) tissues. We found that infection of the MSCs with NDV induced some cell death after 4 days (around 25–40 %, depending on the MSC source, Fig. 2a) and a more pronounced effect after 5 days (data not shown).

We then examined the ability of MSCs to deliver NDV to glioma cells or GSCs using co-cultures plated in transwell plates with a 0.4-μm filter that does not allow cell transfer or by using MSC-conditioned medium.

We first demonstrated that MSCs were able to deliver NDV to co-cultured glioma cells, as indicated by the detection of NDV in the glioma cells using qRT-PCR (Fig. 2b). We did not find a significant difference in the ability of the different MSCs to increase NDV expression in the glioma cells. Similar results were obtained with co-cultured GSCs (data not shown).

As presented in Fig. 2c, co-culturing of glioma cells with MSCs infected with NDV induced a larger degree of cell death in the glioma cells compared to cells directly infected with the same virus titer. Thus, infection of A172 cells with 2 MOI NDV induced about 20 % cell death, whereas co-culturing of A172 cells with NDV-infected BM-MSCs induced over 40 % cell death. Similar results were obtained also with AD-MSCs. Interestingly UC-MSCs infected with NDV induced the largest cytotoxic effect of glioma cells as compared with the other types of MSCs. Similar effects were observed with conditioned medium of the infected MSCs (data not shown). The increased cytotoxic effect of conditioned medium derived from NDV-infected MSCs was also observed in GSCs as compared to their lowered response to direct infection with NDV (Fig. 2d). Similar effects were obtained with measurements of caspase 3/7 activity in both A172 cells (Fig. 2e) and the HF2355 GSCs (Fig. 2f).

### The increased cytotoxic effect of NDV-infected MSCs is mediated by TRAIL

**Fig. 3:**
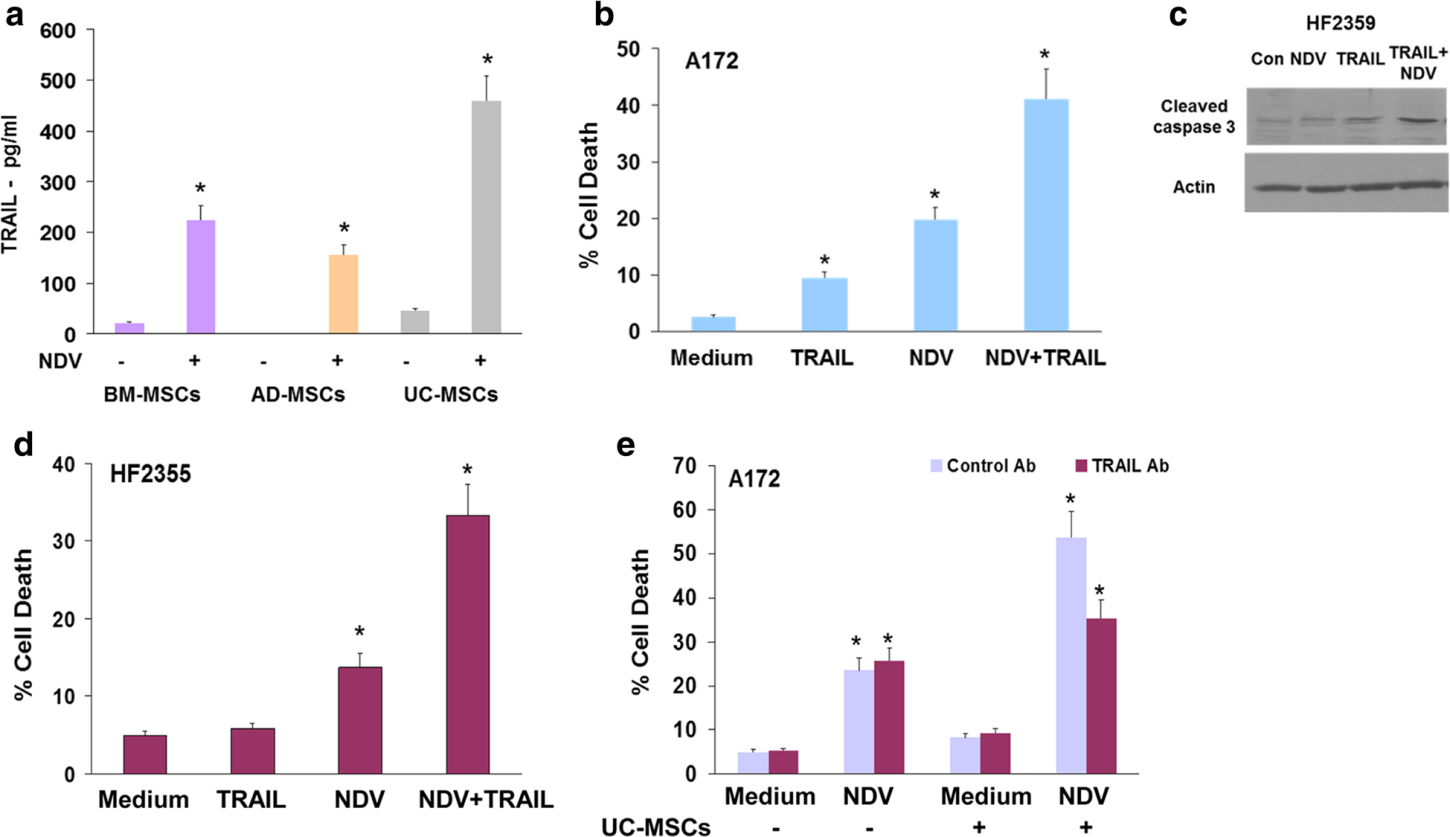
NDV-infected MSCs exert an increased cytotoxic effect on glioma cells and GSCs via the secretion of TRAIL. MSCs were infected with NDV (2 MOI) for 2 days and the levels of secreted TRAIL was determined by ELISA (**a**). Treatment of A172 (**b**), the HF2359 GSCs (**c**) or the HF2355 (**d**) with TRAIL (25 ng/ml) and NDV (1 MOI) induced an increased effect on cell death. The addition of a neutralizing anti-TRAIL antibody (5 μg/ml) prior to NDV infection abrogated the increased cytotoxic effect of conditioned medium derived from UC-MSCs infected with NDV (**e**), whereas it did not affect the cytotoxic effect of NDV infection of glioma cells. The results are presented as mean ± SE and represent three different experiments. ^*^ *p* < 0.001 (control vs. infected cells; NDV + TRAIL vs. NDV and TRAIL; control antibody vs. anti-TRAIL antibody). *AD* adipose tissue, *BM* bone marrow, *GSC* glioma stem cell, *MSC* mesenchymal stromal cells cell, *NDV* Newcastle disease virus, *NSC* neural stem cell, *TRAIL* TNF-related apoptosis-inducing ligand, *UC* umbilical cord

We next analyzed the factors that mediate the enhanced cytotoxic effects of NDV-infected MSCs on glioma cells and GSCs. Using ELISA of infected MSC-conditioned medium we found that control UC cells secreted low levels of TRAIL and that all infected MSCs secreted increased TRAIL levels, with UC-MSCs demonstrating the highest levels (Fig. 3a).

To further examine if the increased TRAIL secretion mediated the enhanced cytotoxic effect of the infected MSCs we first analyzed the combined effect of TRAIL and NDV on cell apoptosis of glioma cells and GSCs. As demonstrated in Fig. 3b, NDV (1 MOI) sensitized glioma cells to the apoptotic effects of low concentrations of TRAIL (25 ng/ml), that by itself induced only a marginal degree of cell death (Fig. 3b). Similarly, NDV also sensitized the GSCs, HF2359 (Fig. 3c) and HF2355 (Fig. 3d) to the apoptotic effects of TRAIL. A neutralizing anti-TRAIL antibody partially inhibited the increased cytotoxic effect of conditioned medium of NDV-infected UC-MSCs on A172 cells (Fig. 3e), suggesting that TRAIL secreted by the infected cells mediated this increased effect. Similar results were obtained with the HF2355 and HF2359 GSCs (data not shown).

In contrast, human astrocytes and the NSCs did not exhibit significant cell death in response to either TRAIL, conditioned medium of NDV-infected UC-MSCs or the combination of both treatments (data not shown).

Altogether, these results implicate TRAIL as an important factor secreted by infected MSCs that can augment the cytotoxic effect of NDV on glioma cells and GSCs.

### Conditioned medium from NDV-infected MSCs sensitize glioma stem cells to γ-radiation

**Fig. 4:**
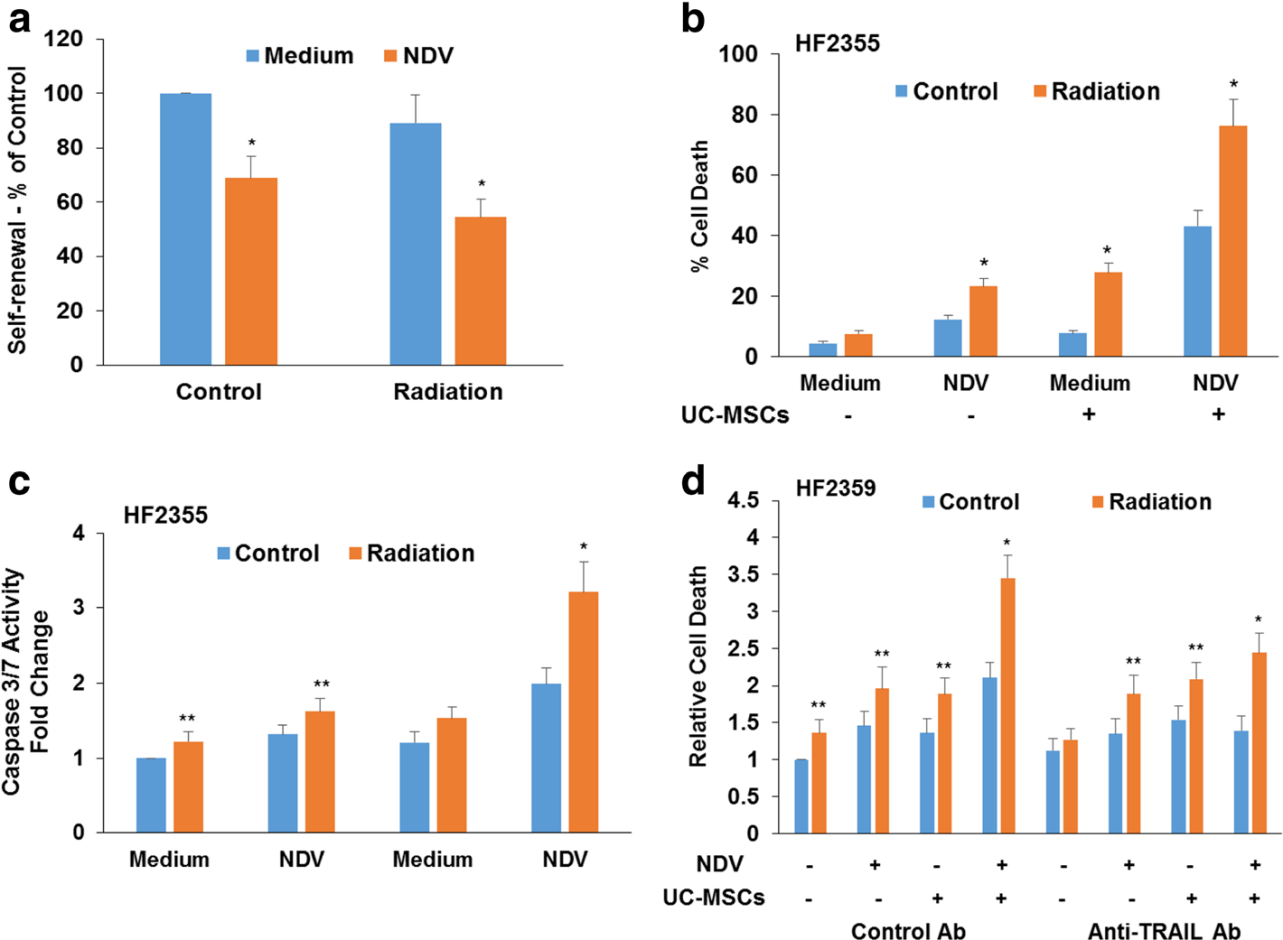
Combined treatment of GSCs with conditioned medium derived from NDV-infected UC-MSCs and radiation exerts a synergistic cytotoxic effect on GSCs. The HF2355 GSCs were infected with NDV and irradiated (3 Gy) (**a**). The self-renewal of the cells was determined after 10 days of treatment. HF2355 GSCs were irradiated in the presence or absence of conditioned medium derived from NDV-infected UC-MSCS (**b**, **c**). Cell death was determined after 3 days using LDH (**b**) or caspase 3/7 (**c**) assays. The role of TRAIL in the increased sensitization to radiation was examined in the HF2359 GSCs. The addition of a neutralizing anti-TRAIL antibody (5 μg/ml) prior to NDV infection and γ-irradiation abrogated the increased cytotoxic effect of the infected UC-MSCs conditioned medium and that of the combined effects of γ-radiation and the conditioned medium. Cell death was measured using LDH assay and data are presented as relative cell death (**d**). The results are presented as the means ± SE and represent three different experiments. ^*^ *p* < 0.001; ^**^ *p* < 0.01. (^*^Control vs. infected cells; radiation + CM of NDV-infected UC-MSCs vs. radiation or NDV alone; control vs. anti-TRAIL antibody; ^**^GSCs treated with UC-MSC CM and NDV infected GSCs vs. untreated cells). *MSC* mesenchymal stromal cells cell, *NDV* Newcastle disease virus, *TRAIL* TNF-related apoptosis-inducing ligand, *UC* umbilical cord

γ-radiation is the first-line treatment for GBM patients; however, GSCs have been reported to exhibit increased resistance to this treatment [49]. Combined treatment of GSCs with γ-radiation and TRAIL induced increased cytotoxic effects [27, 50]. We therefore examined the effect of conditioned medium of NDV-infected MSCs on the response of GSCs to γ-radiation. As presented in Fig. 4a, the HF2355 GSCs exhibited a small decrease in self-renewal in response to γ-radiation treatment (3 Gy) and this response was further increased in NDV-infected cells (1 MOI). Similar results were also obtained with the HF2414 and HF2359 GSCs (data not shown). The effects of γ-radiation and NDV on cell death in these cells were very modest (Fig. 4b). In contrast, we found that a combined treatment of conditioned medium from UC-MSCs infected with NDV and γ-radiation exerted a pronounced effect on cell death as compared to each treatment alone and as was measured by LDH assay (Fig. 4d) and caspase3/7 activity (Fig. 4c). These results suggest that conditioned medium derived from NDV-infected MSCs can sensitize GSCs to the cytotoxic effects of γ-radiation.

We also examined if TRAIL secreted by the NDV-infected UC-MSCs mediated the increased cytotoxic effect of UC-MSC conditioned medium and γ-radiation. Using the neutralizing anti-TRAIL antibody that was employed in the experiments presented in Fig. 3e, we demonstrated that the secretion of TRAIL by the NDV-infected UC-MSCs played at least a partial role in enhancing the response of the GSCs to γ-radiation (Fig. 4d).

## Discussion

In this study, we examined the effects of NDV-infected MSCs from different sources on glioma cells and GSCs and the mechanisms involved in their effects.

NDV has been reported to induce selective apoptosis of various cancer cell lines including glioma [11, 14, 16, 51]; however, its effects on human astrocytes have not been examined. We demonstrated that infection of U87, U251 and A172 cells with NDV induced cell apoptosis already at 1 MOI and that normal human astrocytes were resistant to the cytotoxic effect of NDV even at 10 MOI, further suggesting that NDV effects are tumor cell selective.

GBMs contain a population of GSCs that contribute to therapy resistance and tumor recurrence despite successful surgical removal of visible tumor [52]. Thus, identifying treatments that can selectively target these cells is of utmost importance in the treatment of GBM. Our results show for the first time that NDV can also target GSCs and that although these cells are less sensitive than differentiated tumors cells to the cytotoxic effect of NDV, they exhibit a decreased self-renewal ability when infected with low virus titers. Importantly, also for these cells, the effects of NDV appear to be tumor selective and human NSCs are resistant to NDV infection as indicated in Fig. 1c.

MSCs have been reported to migrate to tumor sites, such as glioma, and to deliver various anti-cancer treatments including oncolytic viruses [31, 32, 53–55]. Our results indicate that MSCs can be also used to deliver oncolytic NDV to glioma cells and GSCs. We found that NDV infected the different MSCs and that these cells underwent apoptosis after 4 days of infection and in response to higher virus titers as compared to glioma cells. These results suggest that NDV-infected MSCs can deliver the virus effectively and can be used safely for the eradication of NDV-sensitive GSCs with no residual presence of MSCs that could theoretically inhibit the local immune response elicited by the infected cells [5, 30].

Interestingly, treatment of glioma cell lines and GSCs with conditioned medium of NDV-infected MSCs induced a larger cytotoxic effect as compared to glioma cells infected with similar NDV titers. The most pronounced effect was obtained with UC-MSC infected cells compared with BM and AD-derived MSCs. We found that the infected UC-MSCs secreted high levels of TRAIL and that treatment of glioma cells with conditioned medium of NDV-infected UC-MSCs in the presence of anti-TRAIL neutralizing antibody abrogated the enhanced cytotoxic effect of the conditioned medium. Moreover, infection of glioma cells and GSCs with NDV sensitized these cells to the apoptotic effect of TRAIL, further supporting our conclusion that this factor mediated at least some of the enhanced cytotoxic effects of the NDV-infected MSCs on glioma cells.

TRAIL has been reported to induce a selective cell apoptosis in tumor cells and has been considered a promising anti-tumor agent [23, 29]. Indeed, multiple studies demonstrated the apoptotic effect of TRAIL on a variety of tumor cells including glioma cells [25–28, 56]. Despite the selective effects of TRAIL on tumor cells there are some cells (in particular cancer stem cells) that exhibit resistance to the apoptotic effect of this ligand [57]. Thus, the current results that demonstrate an increased cytotoxic effect of TRAIL and NDV in both glioma cells and GSCs may provide a mechanism to bypass the relative resistance of GSCs to both TRAIL and NDV. Indeed, a recent study demonstrated enhanced anti-tumor effects of NDV engineered to express TRAIL and IL-2 [58, 59]. The delivery of TRAIL by MSCs engineered to overexpress this protein was recently reported to exert cytotoxic effects in glioma xenografts [60, 61]. Our findings demonstrate a novel approach to increase the secretion of endogenous TRAIL by NDV infection of UC-MSCs, which may further enhance the anti-tumor effects of these cells as was recently reported [62, 63].

Our results of sensitizing GSCs to γ-radiation by the conditioned medium of NDV-infected MSCs demonstrated a mechanism to overcome the resistance of GSCs to radiation, which is one of the first-line treatments for GBM. Indeed, a combined treatment of TRAIL and γ-radiation has been reported to exert a synergistic effect in vitro and in vivo [50]. Importantly, recent studies demonstrated that radiation increased the homing of MSCs to glioma xenografts [50], therefore suggesting that not only NDV-infected MSCs are expected to home to the tumor site more efficiently, but they can also enhance the response of resistant tumor cells to γ-radiation.

Considering the preferential migration of MSCs to tumor sites, it seems reasonable to hypothesize that using MSCs as a delivery system is likely to provide an effective method for the selective targeting of NDV to glioma cells and GSCs. Thus, encapsulating NDV in MSCs can overcome the current limitations in virus delivery and lack of extravasion into the tumor and can shield the viruses from sequestration in the liver and neutralization. Since treatment of GBM patients with NDV seems to be effective in a small fraction of cases [64], it seems reasonable to assume that a more efficient transfer of NDV into the tumor cells may result in a more effective anti-cancer impact for a larger number of patients. Thus, a targeted delivery of NDV by MSCs may represent a substantially more effective approach for transfer of NDV into the malignant cells, rather than relying on random delivery of the circulating viruses.

Preliminary clinical studies suggest that treatment with NDV is safe [16]. Similarly, recent preclinical and clinical studies using both autologous and allogeneic MSCs from various sources indicate that treatment of patients with various neurological and inflammatory disorders is safe and has some therapeutic impact [65–69]. Based on these studies and our results showing the lack of toxicity of NDV or MSCs loaded with NDV on normal astrocytes and NSCs, we expect that treatment with MSCs loaded with NDV is also likely to be harmless against normal neural cells in vivo. Thus, infection of MSCs with NDC may represent a novel approach for the treatment of minimal residual disease and the eradication of GSCs following or along with conventional modalities.

## Conclusions

The infection of UC-MSCs with oncolytic viruses such as NDV can provide a new therapeutic strategy by combining the homing ability of MSCs towards tumor sites and the increased secretion of factors with anti-tumor activity such as TRAIL from the infected cells. Thus, using NDV-infected MSCs is expected to result in targeted delivery of the virus to tumor cells and enhanced anti-tumor effects in both glioma cells and GSCs. In addition, the presence of the infected cells may enhance the local immune response at the tumors sites as was reported for cells infected with other viruses [70]. NDV-infected umbilical cord-derived MSCs may therefore provide a novel effective therapeutic approach for targeting GSCs and GBM and for sensitizing these tumors to γ-radiation.

## Abbreviations

AD: adipose tissue
BM: bone marrow
EGF: epidermal growth factor
FGF: fibroblast growth factor
GBM: glioblastoma
GSC: glioma stem cell
LDH: lactate dehydrogenase
MOI: multiplicity of infection
MSC: mesenchymal stromal cells cell
NDV: Newcastle disease virus
NSC: neural stem cell
TNF-α: tumor necrosis factor alpha
TRAIL: TNF-related apoptosis-inducing ligand
UC: umbilical cord
